# Bibliometric‐Based Analysis of Global Trends and Collaborative Networks in Plant Genetic Engineering (1994–2024)

**DOI:** 10.1111/pbi.70550

**Published:** 2026-01-19

**Authors:** Tongxiao Xu, Teng Wang, Cong Zhang, Yuan Cao, Xiaoyun He

**Affiliations:** ^1^ Key Laboratory of Precision Nutrition and Food Quality, Key Laboratory of Functional Dairy, Ministry of Education, College of Food Science and Nutritional Engineering China Agricultural University Beijing China; ^2^ Library China Agricultural University Beijing China; ^3^ Information Research Center of China Agricultural University Beijing China; ^4^ Key Laboratory of Safety Assessment of Genetically Modified Organism (Food Safety) Ministry of Agriculture and Rural Affairs of the P.R. China Beijing China

**Keywords:** Bibliometrics, CRISPR, genetic engineering, GM

## Abstract

Agricultural sustainability faces serious challenges from population growth, climate change and ecological degradation. Genetic modification (GM) technology can be regarded as a precise extension of the Green Revolution, aiming to balance yield enhancement with ecological integrity through biotechnology. To systematically examine global trend, this study conducts a bibliometric analysis using worldwide literature data from 1994 to 2024. The findings reveal a dual‐core structure of international collaboration, centered on China and the United States. The United States is closely connected with Korea, Japan and the United Kingdom, forming a high‐density cluster, while China engages with emerging regions in Southeast Asia and Africa through the Belt and Road Initiative. This initiative is intended to strengthen China's influence and is accompanied by the proliferation of technology in countries less endowed with resources. The technology lifecycle has been evolved through three distinct phases. Initially, the process of Agrobacterium‐mediated transformation in tobacco plants was carried out, marking the beginning of transgenic development. This was followed by the implementation of RNA interference (RNAi) technology to silence multiple genes. Finally, a breakthrough happened through the development of CRISPR‐Cas9 genome editing technologies. The analyses conducted in this study demonstrate the preponderance of CRISPR in contemporary research, thus suggesting that the industry places a premium on technological refinement. Hence, the future technological trajectory is predicted to focus on germplasm digitization, multi‐gene editing, intelligent breeding and synthetic biology. Transgenic technology will serve as a foundational support for achieving sustainable food security in the forthcoming second green revolution.

## 1 Introduction

The significance of agriculture to civilisation is well documented and remains vital. Food is a strategic resource for national livelihood, economic security and public sustenance (Gao and Cui 2023). In the early 1970s, a period of significant global economic and political turbulence, grain stocks declined precipitously, leading to a sharp price escalation. This precipitated a series of food shortages across numerous countries, leading the international community to accord food security greater systematic attention (Khanna 2021). The Food and Agriculture Organisation of the United Nations (FAO) first proposed the concept of food security in 1974 (Maxwell 1996). Since then, the definition has undergone continuous revision and evolution. The current understanding of food security is a guarantee that sufficient food is available and affordable to all people at all times for their survival and health (Hajian and Jangchi Kashani 2021). It involves producing sufficient quantities of food, stabilising the food supply to the maximum extent possible, and ensuring food is available to all who need it (Habib et al. 2025).

Agricultural systems face mounting pressures from multiple stressors (Liang et al. 2024). Global population growth and dietary changes require increased food production amid natural resource constraints and scarcity (Godfray et al. 2010; Ma et al. 2018). It is evident that frequent natural disasters, pandemics and conflicts driven by climate change are weakening food systems (Tilman et al. 2011). In the context of the global epidemic of Coronavirus Disease 2019 (COVID‐19), the primary objective of any nation must be to ensure its own self‐sufficiency (Dodd et al. 2021). Moreover, the Green Revolution in agricultural technology, which spread globally in the 1940s and 1970s, while promoting high‐yield crops, fertilisers, irrigation and mechanisation, greatly enhanced food production, guaranteeing food security and reducing hunger (Fiaz et al. 2024). However, it also introduced long‐term challenges, including overuse and misuse of pesticides, contamination from heavy metals and other environmental and technological risks (Dash and Rai 2022; Liu et al. 2022). Achieving and maintaining high crop yield is contingent upon the increased utilisation of fertilisers. The escalating costs of fertilisers and other inputs, in conjunction with the fragmentation of land ownership, have precipitated a decline in farmers' incomes. Consequently, a significant proportion of farmers have been compelled to seek alternative economic activities beyond agriculture (Evans and Lawson 2020; Hamdan et al. 2022).

Genetic engineering was born to address the problems left over from the first Green Revolution (Ma et al. 2018). It is an extension of traditional breeding techniques by accelerating trait improvement through precise gene editing and other methods, achieving the dual goals of increasing yields and reducing harm, adding new capabilities to cope with climate change and shortening the breeding cycle (Gaudin et al. 2013; Krichevsky et al. 2012). However, the development of transgenic plants has been a controversial issue, with differing views and opinions (Ahmad et al. 2023). Genetically modified plants are widely used in some countries and regions, while in others, they are still under strict control and regulation (Sang et al. 2013). Early examples of genetically modified plants include virus‐resistant tobacco, genetically modified rice and genetically modified tomato. The development of genetically modified plants is now highly diverse, and the current global area of genetically modified crops exceeds 190 million hectares, with 79% of cotton and 74% of soybeans being genetically modified varieties (Qaim 2016). Several genetic modifications have been made to crops to improve yield, quality and resistance to various agricultural challenges. Rice is one of the model plants used in genome research. It has a small genome of around 430 million base pairs, which was sequenced in 2002. It has a clear genetic background that facilitates gene editing and functional verification. Meanwhile, rice tissue culture technology is well developed and highly efficient, providing a technical foundation for genetic modification. Advances in gene editing technology, particularly CRISPR, have made it easier to develop maize varieties with specific, targeted modifications (Barrangou 2023; Ishino et al. 2018). Unlike traditional genetic modifications, CRISPR technology allows for more precise and less controversial changes to the maize genome, including increased resistance to pests and diseases, as well as improved nutritional value of maize (Ma et al. 2014; Pan et al. 2021). Improved resistance to pests is a key focus of transgenic plant development, and one of the most common transgenic plant traits is resistance to specific pests, particularly the European corn borer and rootworms, which is achieved through the introduction of genes from the bacterium *Bacillus thuringiensis* (Bt). Bt maize significantly reduces the need for chemical insecticides and improves yields (Guan et al. 2023; Liu, Guo, et al. 2020). An advanced form of the Green Revolution, genetic engineering uses traits like drought resistance to boost yields and protect the environment, yet its global use is still widely contested (Tabashnik and Carrière 2004).

Innovation ecosystem theory provides a framework to quantify complex ecosystems, encompassing key risks like collaboration dynamics among stakeholders and the challenge of technological complementarity. Conventional qualitative analyses have limitations from subjective interpretation. Conversely, bibliometrics can quantify knowledge flow paths, collaboration patterns and risk nodes within an ecosystem. Methods include collaborative network analyses and thematic clustering. This quantification helps address the limitations of traditional case studies, which are one‐sided (Adner 2006). Traditional review methods also struggle to capture contextual shifts in the innovation ecosystem. For example, assessing the significance of technological or market factors in the development of genetic modification is complex, and conventional literature reviews cannot adequately address this. The integration of bibliometrics with content analysis facilitates the systematic identification of the evolutionary trajectory of research topics, the detection of the temporal stage of technological shifts, the revelation of the limitations of theoretical applications in diverse contexts, and the provision of data support for theoretical innovations (Carayannis et al. 2013).

Notwithstanding the proliferation of research endeavours concerning transgenic plant technology, systematic bibliometric analyses remain deficient in two pivotal domains. On the one hand, international collaboration patterns are worthy of consideration; previous studies have lacked a quantitative framework for mapping the evolving global knowledge networks, particularly with regard to the role of emerging economies such as China in the reshaping of collaborative dynamics. Conversely, technological generational shifts have been observed. The extant literature has not yet fully captured, through a longitudinal lens, the paradigm shifts in transgenic research (e.g., from classical transgenic overexpression approaches to CRISPR‐driven precision editing). This study addresses these gaps by integrating bibliometric tools to analyse the structural evolution of global research networks and identify generational breakpoints in technological innovation.

## 2 Materials and Methods

### 2.1 Data Sources and Collection

The Web of Science Core Collection (WOS) is a premier global repository of scientific knowledge, containing bibliographic elements and citation data for scholarly publications. These include title, year of publication, country, institution, keyword distribution within the field of study, and citation links. In addition, the database's compatibility with numerous analytical tools makes it a popular choice for interdisciplinary bibliometric analyses. Our study included all English‐language research‐based articles and reviews on transgenic plants from 1 January 1986 to early December 2024 in the Web of Science Core Collection. The search approach employed is outlined as follows:

((title = (“genetically modified”) AND title = (plant) AND title = (“gene technology”)) OR (abstract = (genetically modified) AND abstract = (plant) AND abstract = (gene technology))).

And the ‘plants’ refer to genetically modified plants, including, but not limited to, rice, maize, cotton, soybeans and tomatoes. For further details on search formulas, consult the Appendix S1 section. Full bibliographic features and citation information were independently searched and downloaded on 6 December 2024 from publicly accessible databases. The findings for 2024 are based on data available up to December 6, which we believe accurately reflect the year's trends. This study did not involve animal testing or human participants; therefore, ethical approval was not required.

### 2.2 Methods of Analysis

**FIGURE 1:**
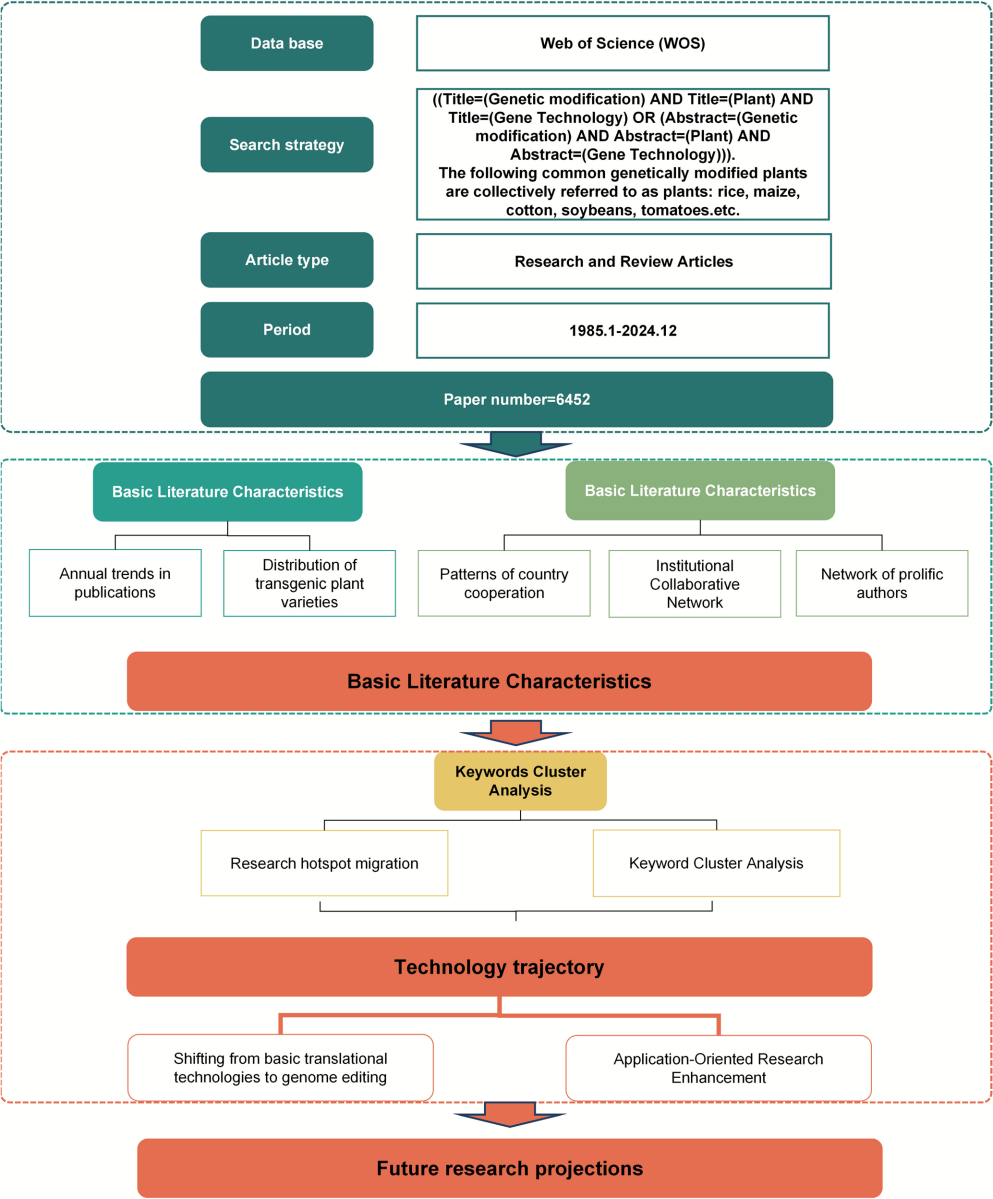
Technology roadmap.

To map collaborative networks in transgenic plant research, we used CiteSpace 6.3.R1 to analyse references with Citation Bursts and VOSviewer 1.6.20 to organise, statistically analyse, and visually present data on literature authors, journals, institutions, countries and keyword clusters to create a comprehensive network. Additionally, Microsoft Office Excel 2019 was used to conduct quantitative analysis of the publications. The technical approach is shown in Figure 1.

**FIGURE 2:**
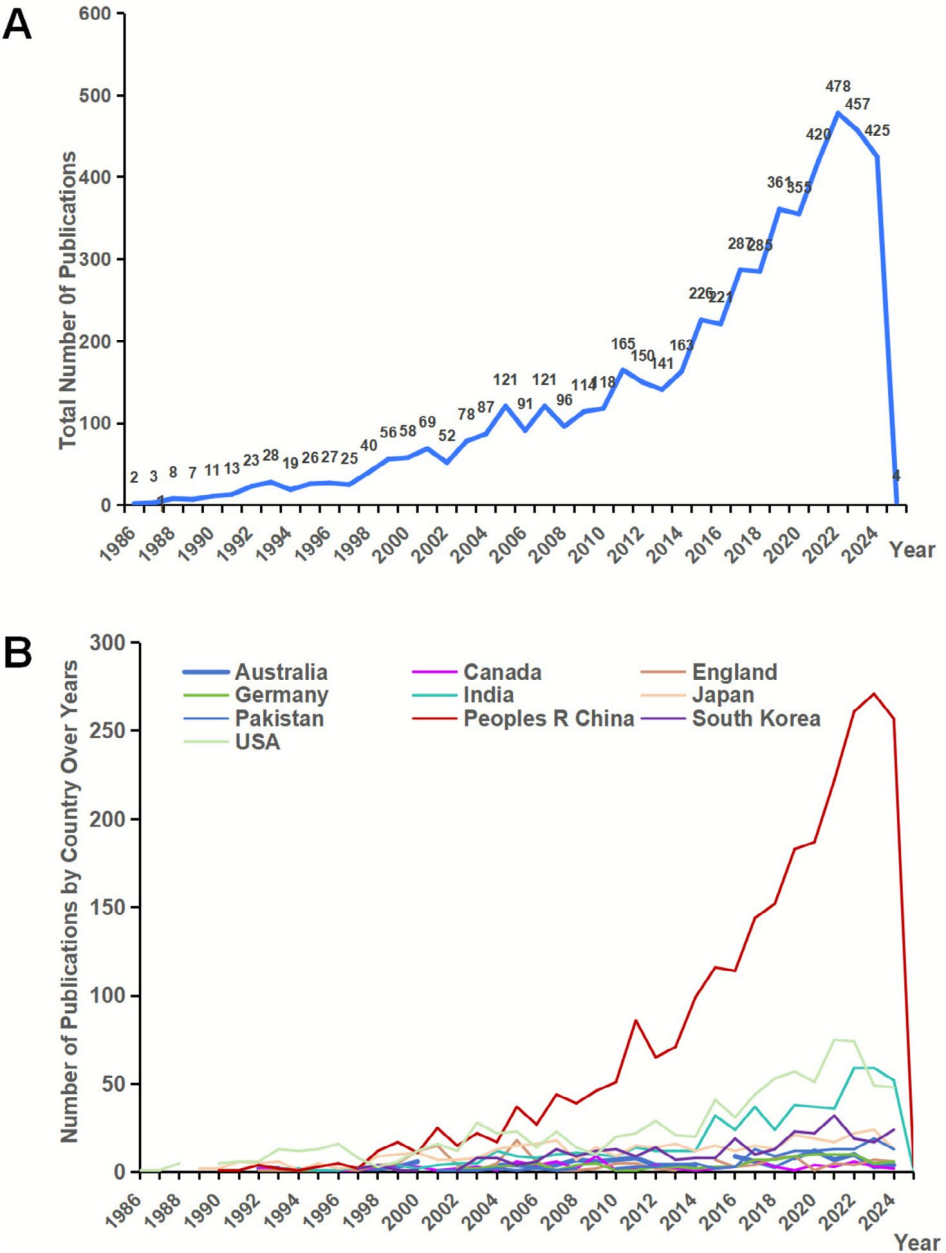
Annual publication of literature related to transgenic plants and country of publication. (A) Annual publication of literature related to transgenic plants. (B) National annual publication of literature related to transgenic plants.

The earliest relevant literature dates to 1985, with few sporadic publications through 1993 representing a pre‐commercialization phase. In 1994, after the first commercial crop, growth stabilised and systematic development began. For consistency, this study defines 1994–2024 as the main analysis period. Early years are shown in some charts (e.g., Figures 1 and 2) to illustrate the technology's origins and full trajectory.

## 3 Results

### 3.1 Characteristics of the Underlying Literature

#### 3.1.1 Annual Trends in the Issuance of Communications

Our final integrated dataset covers 6452 academic papers on transgenic plant research. The analysis shows that the average publication year of these papers is about 10 years ago, reflecting the continuous academic accumulation and attention in this field. From an impact perspective, this paper averaged 46 citations, indicating that the research field as a whole has considerable academic value and influence. To reveal the long‐term evolutionary trend in this field, Figure 2A highlights the annual publication numbers for transgenic plant research globally over the past nearly four decades (1985–2024) and focuses on the top 10 countries by publication numbers. In terms of country distribution, China absolutely dominates the scientific output in this field, and its research has shown a sustained, significant increase in the number of annual publications, which have jumped and stabilised at more than 100. Over nearly three decades, China's total number of publications has approached 3000, far ahead of other countries (Figure 2B). The countries following China are the United States and India, with a total of 904 publications in the United States and 548 in India in the last 40 years. Unlike the strong growth in China, the number of annual publications in these two countries has remained consistently below 100. However, research activity in the United States and India has also demonstrated a general upward trend, albeit with some minor fluctuations.

#### 3.1.2 Species Research Distribution

**FIGURE 3:**
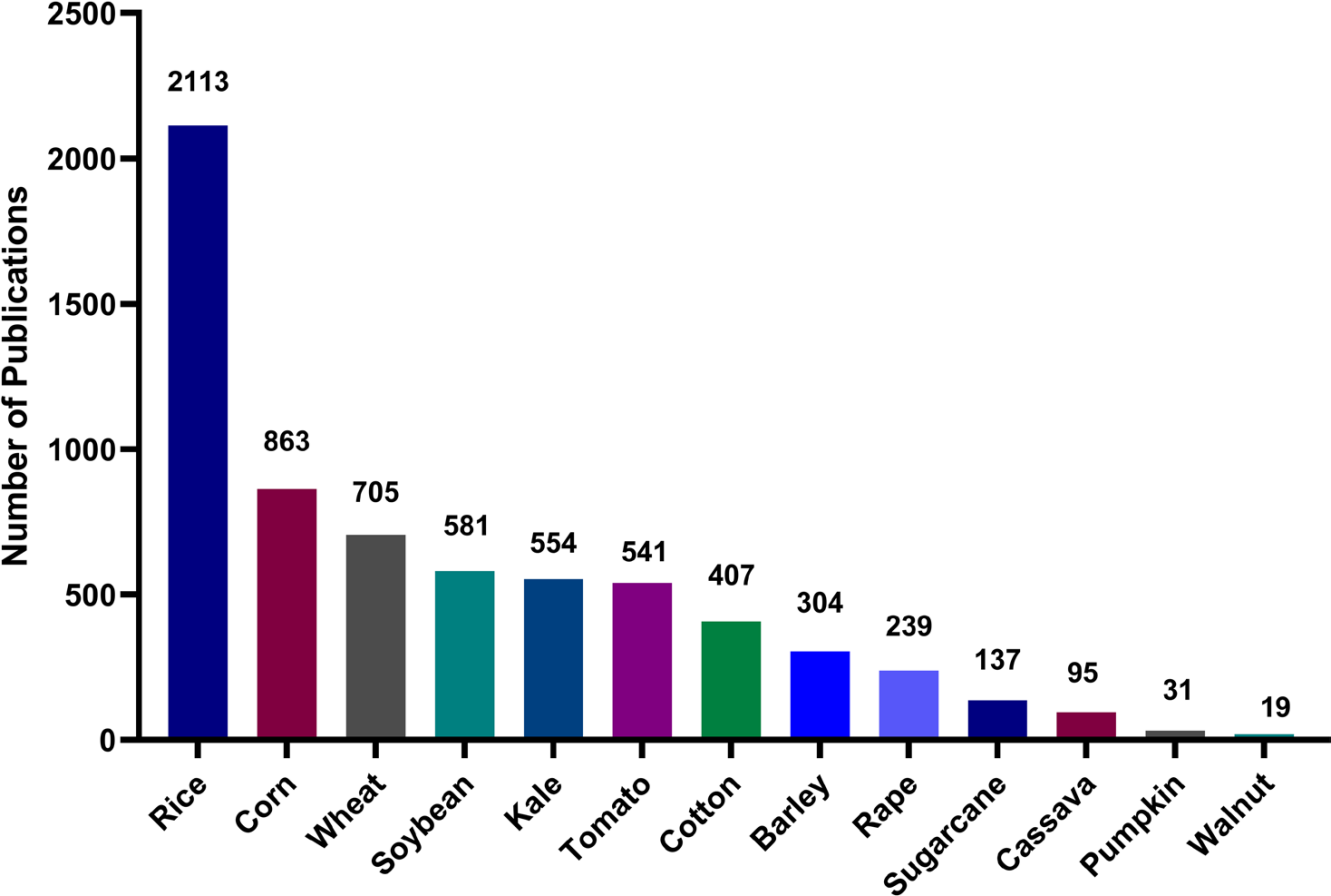
Histogram of the total number of papers.

Figure 3 clearly reveals the distribution of academic attention to different crop varieties in transgenic plant research. In terms of total publications, rice overwhelmingly dominates, accounting for 57.5% of research on the three major staple crops (rice, maize and wheat) and 2113 relevant papers. This may be because rice is a staple crop in Asia and can drive priority research and development for traits such as insect and herbicide resistance (Tilgam et al. 2021). In contrast, maize (863) and wheat (705) ranked second and third, with the combined literature still significantly lower than that of rice, highlighting rice's absolute dominance in transgenic research. It is speculated that this is due to the small genome and mature genetic transformation system of rice (Banakar and Wang 2020), which are conducive to gene function verification and technology iteration (Liu et al. 2024). By comparison, maize's genome (2.3 Gb) is five times larger than rice's, and wheat's genome (16 Gb) is 37 times larger. This genome size difference makes genetic manipulation in maize and wheat more challenging (Yu et al. 2021; Majumder et al. 2021). Consequently, the genetic transformation rate of wheat has long remained below 5%, whereas rice's stable system exceeds 30% (Li et al. 2022). This also increases the difficulty of research, resulting in a lower number of publications compared to rice.

**FIGURE 4:**
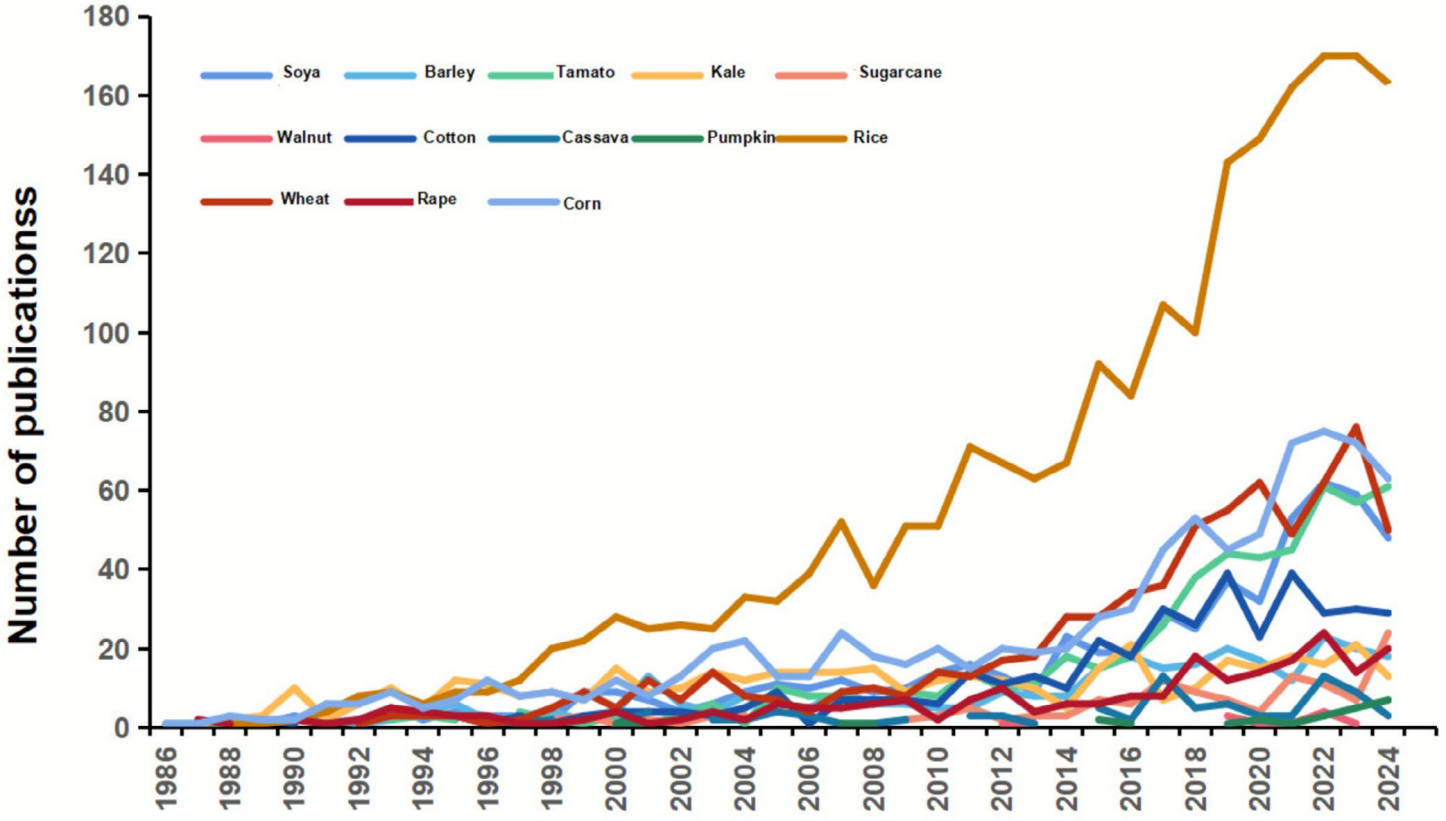
Trend plot showing years of publication for different plants.

This distribution pattern aligns with the temporal trend in Figure 4. Figure 4 also shows that annual publication volumes for the three major staple crops trend upward, with rice showing the fastest growth. Notably, its annual publication count exceeded 100 for the first time in 2017 and has continued to grow since then. This rapid increase in rice publications may be due to advances in CRISPR technology. Emerging in 2013, CRISPR quickly moved from mechanistic research to tool development, enabling widespread use after 2015. This technological progress supported rapid growth in research from 2017 (Barrangou 2023; Pan et al. 2021). In contrast, the annual publication volumes for maize and wheat have stayed below 100, with only steady growth. One reason for the surge in rice research could be that rice breeding was a key speciality in China's 13th Five‐Year Plan (2016–2020), which provided policy support and resources (Center for Security and Emerging Technologyegic Emerging 2016). It is speculated that this may have accelerated the output of rice research through policy orientation and resources. The adoption of CRISPR in staple crops like rice and maize was also aided by established transformation systems and the crop's importance to food security. For cash crops such as soybeans and cotton, studies also increased, illustrating how gene‐editing tools have reduced reliance on traditional model plants and broadened the focus to food security and sustainable agriculture.

### 3.2 Academic Impact Analysis

#### 3.2.1 Patterns of Country Cooperation

**FIGURE 5:**
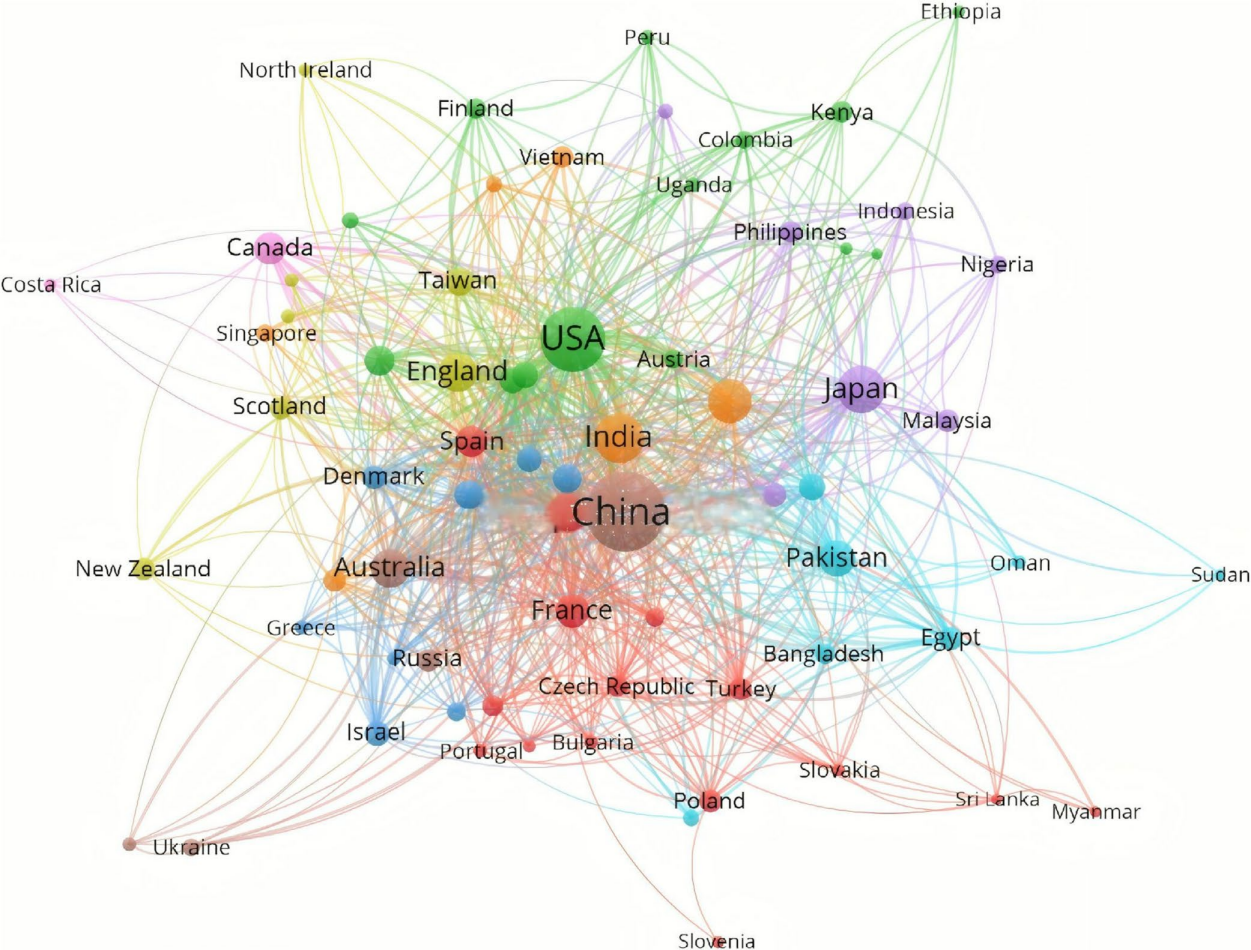
National cooperation networks in the field of transgenic plants over the past three decades.

To analyse the pattern of scientific resource allocation and the distribution of academic discourse in the global transgenic plant research systematically, the dataset used in this study includes publication data from 603 institutions in 78 countries. In the past three decades, the leading countries in the GM plant‐related literature were China (*n* = 2613, 71.2%), the United States (*n* = 904, 24.63%) and India (*n* = 548, 14.93%). Among these, China dominated the literature output, with 1.8 times the publications of the USA and the third‐place country combined, a finding that highlights the rise of Asian countries in this field. Figure 5 illustrates the network of national collaborations in the field of transgenic plants over the past three decades. China and the United States are at the centre of this network, demonstrating their dominant dual roles in transnational knowledge flows.

#### 3.2.2 Institutional Collaborative Network

**FIGURE 6:**
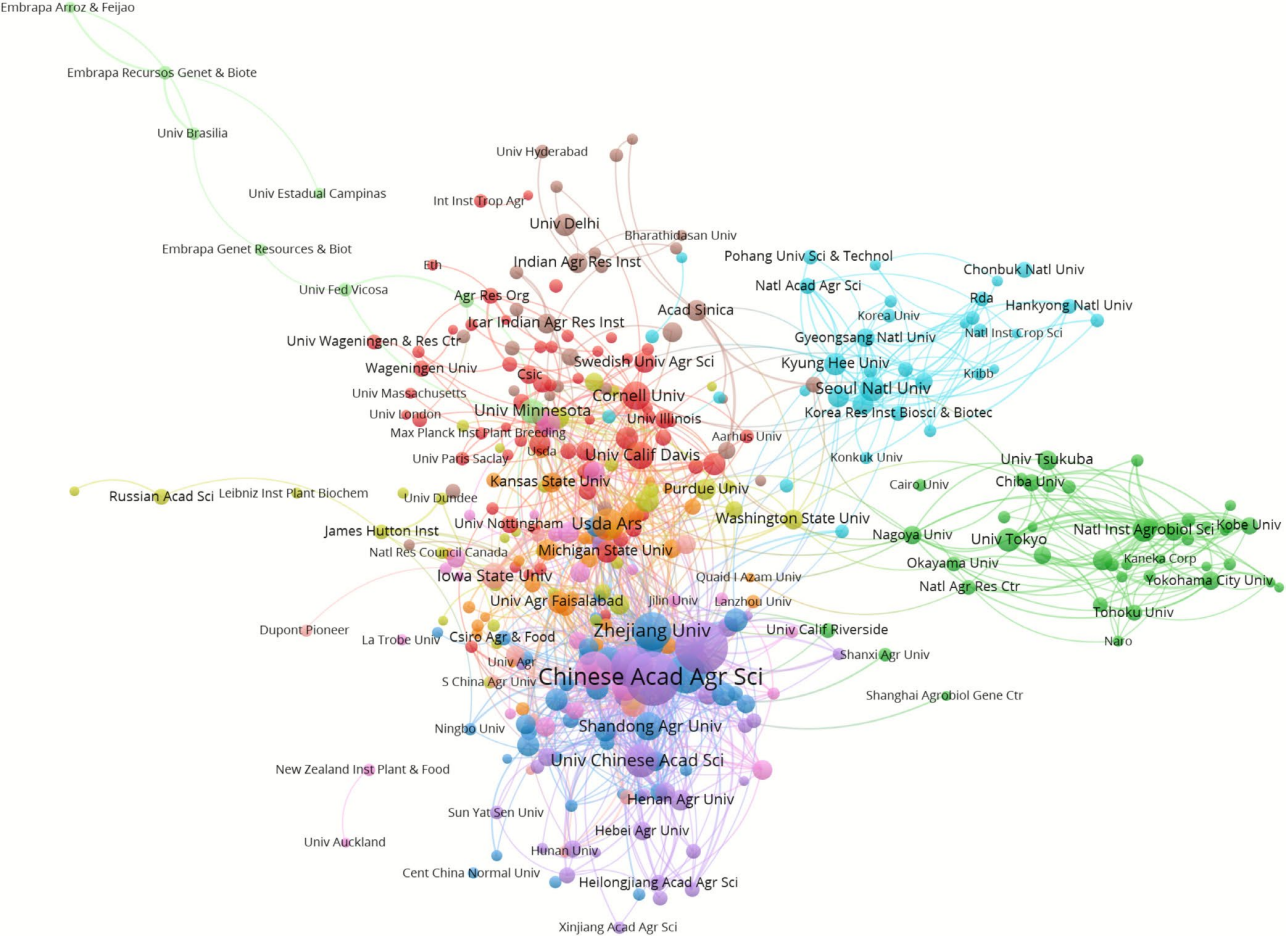
Institutional cooperation networks in transgenic plant research over the past three decades.

Figure 6 presents the collaboration visualisation map of the top 50 most frequently cited agricultural research‐related institutions. Different coloured nodes represent the proximity of collaboration: for example, institutions with similar research directions and with frequent collaborations and exchanges are shown in similar colours, a method that demonstrates visually the collaboration links between institutions. The node size is positively correlated with the number of citations, and larger nodes, such as Chinese Acad Agr Sci and Zhejiang Univ, indicate that the research results of these institutions influence the field strongly and are cited frequently. The lines are a reflection of the intensity and frequency of institutional cooperation: lines that are thicker and denser indicate closer the cooperation. The distribution indicates that global agricultural research institutions collaborate across geographical areas to form a multicentre network. This finding demonstrates that the global agricultural research field has promoted knowledge‐sharing and technological innovation, and has developed a transregional scientific research collaborative network mainly through Embrapa‐related institutions in Brazil, many agricultural colleges in India, research institutes in South Korea, and many agricultural research forces in China via projects, academic exchanges and other links. This network promotes knowledge‐sharing and innovation in the agricultural research, helps to solve common problems in agriculture, and promotes the development of agricultural science and technology.

#### 3.2.3 Prolific Authors Network

**FIGURE 7:**
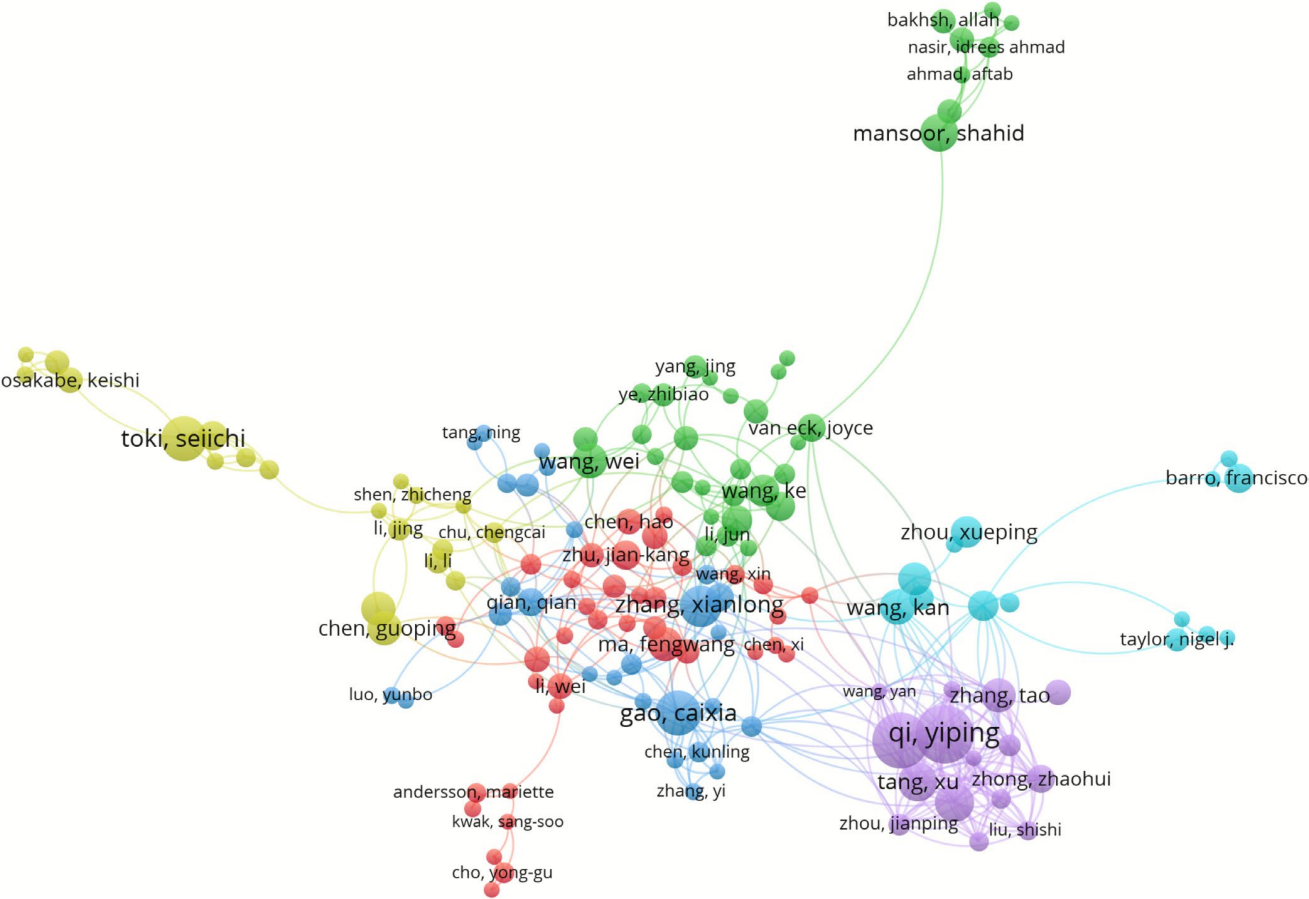
Author collaboration network in the transgenic plants research over the past three decades.

Institutional collaboration is essentially a network of trust between core authors (Ninkov et al. 2021). To shed light on resource‐sharing and talent collaboration for knowledge production in research networks, 29 857 authors were included in the data and analysed. Over the past 30 years, several prolific authors have emerged in this field, including 22 authors who have published 20 or more papers. These individuals have established a strong network of scholarly communication and collaboration. Figure 7 shows a visual network diagram of the top 50 authors over the last 30 years, with thicker lines indicating closer collaborations and darker colours indicating earlier average publication times. The colour coding in the figure reflects the strong links within a particular research area. The greatest number of articles were published by Qi, Yiping (*n* = 41, 0.60%) and Zhang, Yong (*n* = 39, 0.57%). The H‐index of 28 highlights Qi, Yiping's large academic impact. In addition, researchers such as Gao Caixia, Wang Yanpeng, Voytas Daniel F., Liu Jinxing, Li Boshu, Zheng Xuelian, Tang Xu, Chen Kunling, Yang Bing, Li Yan, Qiu Jinlong, Lippman Zachary B. and Zhang Yi, each of whom worked very closely with the others, possess some authority in the field.

To reveal the stratification of academic influence and generational turnover among the prolific authors in the transgenic plants research, we listed the quantitative assessment indices of prolific authors with bibliometric core parameters in Table 1. Top scholars (e.g., Gao Caixia) led the technological breakthroughs with original innovations, while technology application experts such as Wang Yanpeng (3703 total citations) drove the diffusion of the results. Moreover, the proportion of new‐generation authors (e.g., Li Boshu, with an average year of publication of 2022.2) increased drastically to 40%. Additionally, the standardised citation impact of these authors surpassed that of the earlier group of researchers, which indicates that the generational transmission of the field is complete. This pattern drives the evolution of global academic power towards multipolarity, with traditional European and American institutions and emerging research forces working together to reconfigure the innovation ecosystem.

**TABLE 1:** Collaborative assessment of prolific authors in the field of transgenic plants over the last three decades.

Author	Weight <Citations>	Weight <Norm. citations>	Score <Avg. pub. year>	Score <Avg. citations>	Score <Avg. norm. citations>
Li, Boshu	489	73.4	2022.2	97.8	14.7
Wang, Yanpeng	3703	136.8	2019.2	336.6	12.4
Yang, Chao	187	47.2	2021.5	31.2	7.9
Gao, Qiang	319	46.5	2014.2	53.2	7.8
Chen, Qi‐Jun	2070	37.8	2020.2	414.0	7.6
Chen, Letian	1699	37.8	2019.6	339.8	7.6
Liu, Jinxing	2965	75.4	2018.9	296.5	7.5
Wang, Zhi	1559	34.8	2021.4	311.8	7.0
Gao, Caixia	6195	202.2	2019.1	199.8	6.5
Lin, Qiupeng	991	32.4	2019.6	198.2	6.5

### 3.3 Knowledge Evolution Pathways

#### 3.3.1 Keyword Cluster Analysis

**FIGURE 8:**
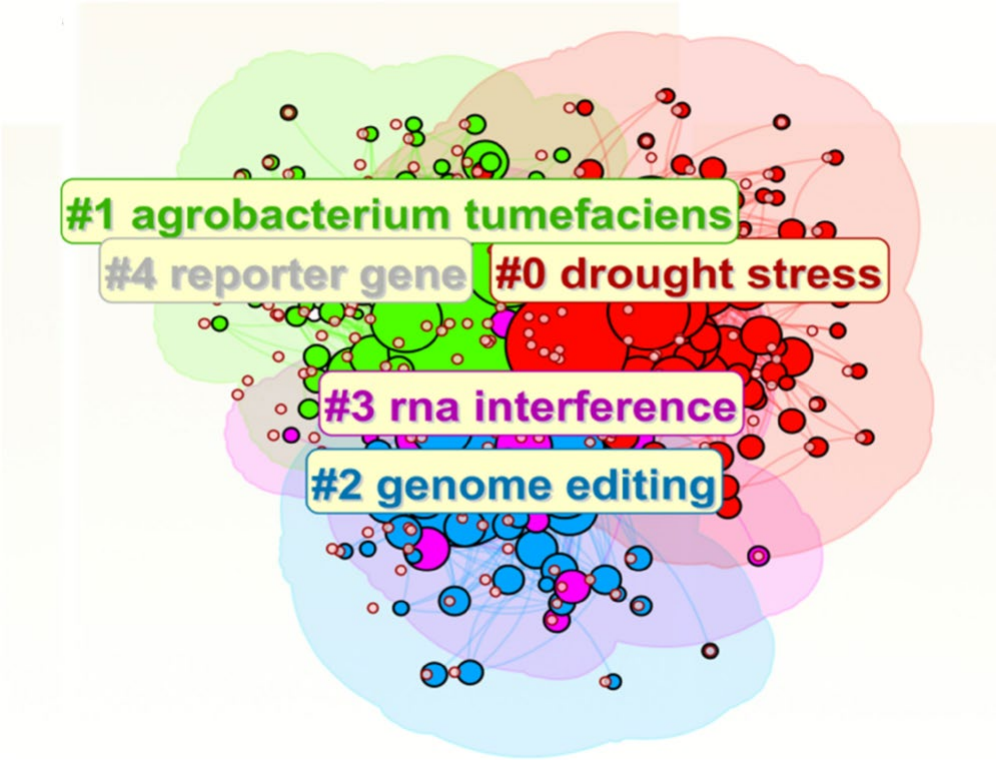
Visualisation of keyword clustering in the field of transgenic plant research over the past three decades.

The research trajectories of high‐yield authors were analysed in the previous section. Because the keywords directly drive technical conceptual evolution, the keyword network in the transgenic plant research was visualised to highlight the correlation and degree of research intensity between different research topics. Individual keywords are represented by nodes of different colours and sizes in Figure 8, whereas the lines connecting the nodes indicate the relationships that co‐occur between these themes. Among them, Agrobacterium is often used as a tool for plant genetic transformation, helping to introduce exogenous genes into plant cells (Krenek et al. 2015). Reporter genes can reflect the regulation of gene expression intuitively and assist in the study of gene function (Cohen et al. 2022). Drought stress is a common form of abiotic stress in plants, and the investigation of its response mechanism and drought‐resistant genes is beneficial for the cultivation of drought‐resistant crops (González 2023). Drought stress was the primary research focus within abiotic stresses, although a broader range of stress types was found. RNA interference (RNAi) can suppress the expression of genes through specific RNA molecules and is widely applied to improve crop genetics (Hung and Slotkin 2021). Genome editing techniques are widely used in the field of genomic editing. These keywords reflect the research hotspots in this field, and by analysing the co‐occurrence of keywords, we can help shed light on emerging trends, explore the thematic connections and guide future research.

#### 3.3.2 Research Hotspot Migration

**FIGURE 9:**
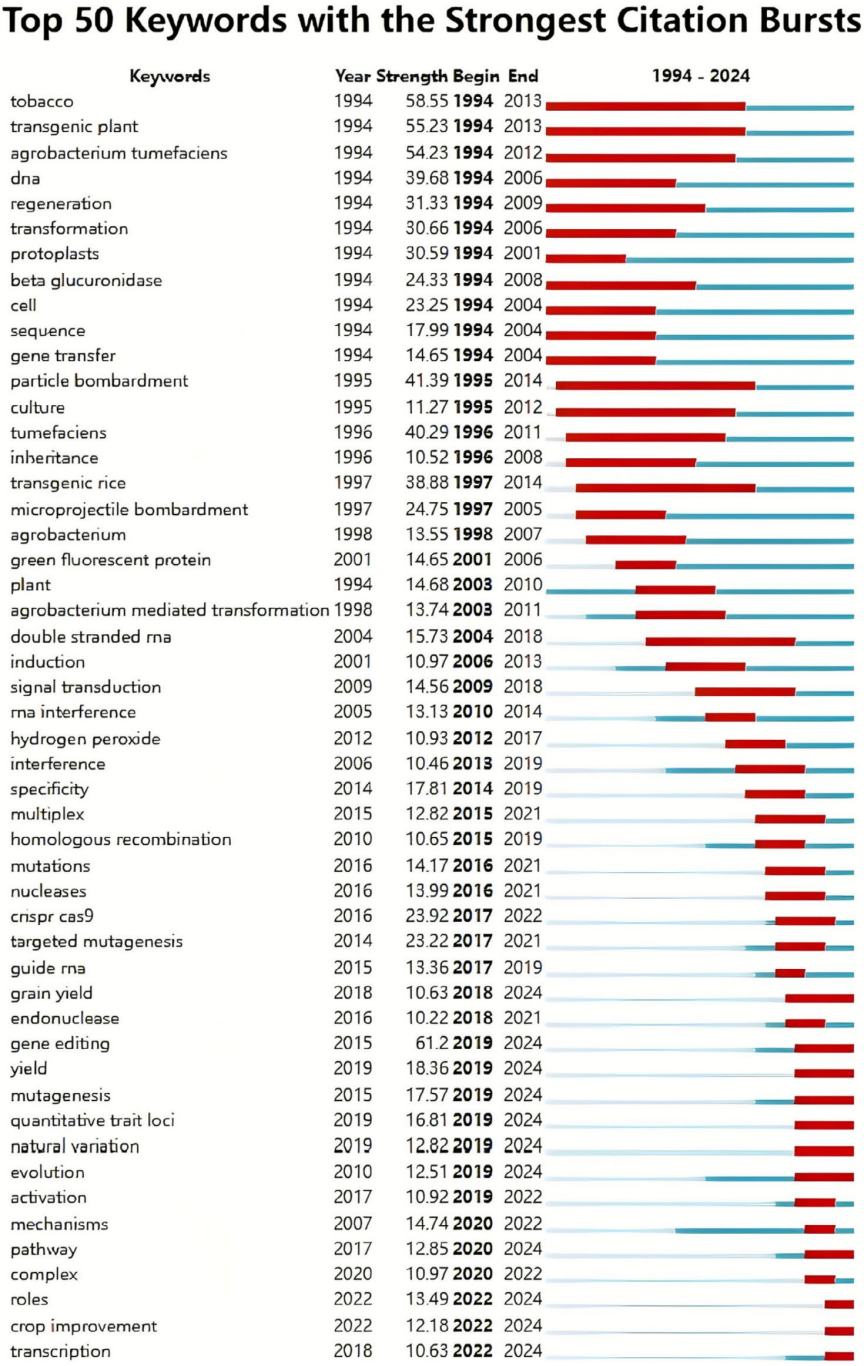
Top 50 keywords with the strongest citation surge in the dataset.

To more clearly track technological inflexion points and changes in research hotspots, we list the top 50 keywords that burst in the dataset (Figure 9) to indicate the sudden increase in keyword citations. In the early years (1994–2006), the most prominent keywords included ‘tobacco’, ‘transgenic plants’ and ‘agrobacteria’, with ‘tobacco’ reaching its highest citation intensity in 1994. As a model plant, ‘tobacco’ was the most commonly used and mature research system in early gene transformation experiments, and its citation explosion point (1994) marked the establishment and widespread application of this technology platform (Fethe et al. 2014). As genetic editing techniques advanced and research priorities shifted towards applied agriculture, keywords related to major crops such as rice, maize, soybean and cotton gradually gained prominence, reflecting the field's transition from model systems to economically important species. *Agrobacterium tumefaciens* was the most important tool for plant genetic transformation at that time (Ravanrouy et al. 2021). The core objective of this stage was to achieve the stable integration and expression of exogenous genes in plants and to verify the feasibility of the technology. As the basic transformation technology matured, the research focus shifted inevitably to more challenging and practical directions. This shift directly drives the keyword evolution in the later phase (2007–2024) (Fethe et al. 2014). The keywords that exhibited the most significant growth from 2007 to 2024 include ‘gene editing’, ‘gene editing technology’, ‘yield’, and ‘crop improvement’. The analysis of these high‐frequency outbreaks and frequently cited keywords provides a deeper understanding of the changing trends and focus of transgenic plants and reveals that the transgenic plants research focus has shifted gradually from the basic establishment of transgenic technologies to more complex genome editing and crop improvement strategies and to the subsequent application of technologies to solve the core problems.

**FIGURE 10:**
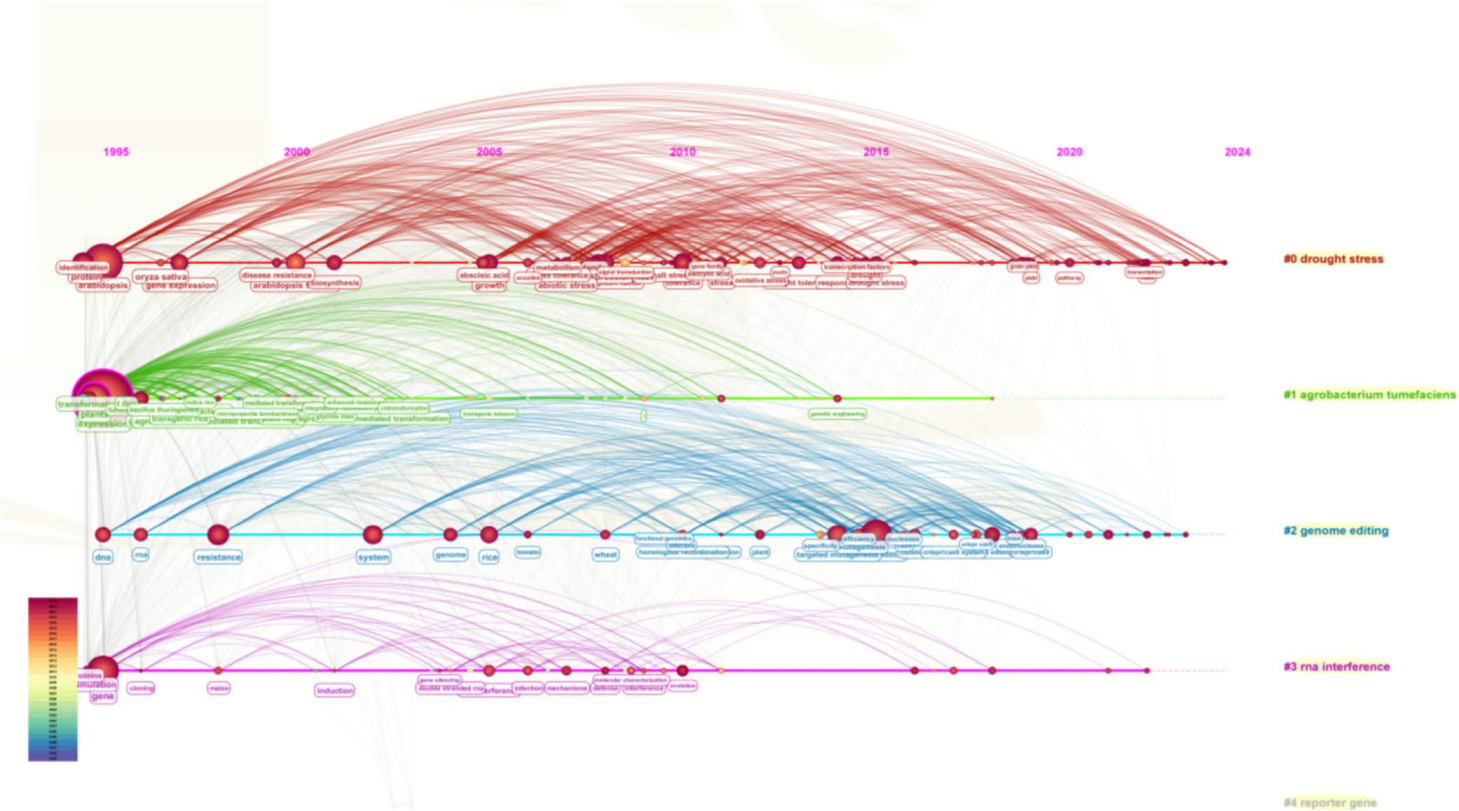
Timeline visualisation of keyword clustering in the field of transgenic plant research over the past three decades.

To illustrate the phased evolution of transgenic plant research technologies more intuitively, we constructed a keyword temporal network diagram (Figure 10). This diagram not only objectively identifies research themes but also dynamically reveals the emergence, persistence, and succession of different technological themes, thereby addressing the limitations of a static clustering analysis. The node sizes indicate the citation frequencies, their position indicates the number of citations, and the line thickness between nodes indicates the correlational strength. The network, which includes 597 nodes and 1814 lines, has a modularity greater than 0.3 (*Q* = 0.326), which indicates a strong cluster structure, and a contour (*S* = 0.4537) that delineates clearly between the four identified thematic clusters. These four thematic clusters represent research hotspots. The node representing ‘drought stress’ (#0) was consistently prominent and brightly coloured from 1995 to 2024, a finding that highlights this as an area of continued interest. These findings indicate that tolerance to abiotic stress represents a persistent core driver of transgenic technology development. The research related to ‘genetic transformation’ (#1)—including terms such as ‘cloning’, ‘ransgenic plants’ and ‘Agrobacterium‐mediated transformation’—reveals that these are areas of continued interest. This finding underscores that a highly efficient genetic transformation system constitutes the foundational technology underpinning the entire field's advancement, as such a system has continuously remained a research focal point. Moreover, ‘genome editing’ (#2) and ‘RNA interference’ (#3) have gained sustained attention as research themes over a period of three decades, and in recent years have again become research hotspots. These findings indicate that these topics represent the most active technological frontiers at present, signifying a shift in the field from traditional transgenic techniques towards a new paradigm of precise and efficient gene regulation. Moreover, the lines connecting the nodes indicate that these themes did not evolve in isolation. For example, the strong link between ‘genome editing’ (#2) and ‘gene transfer’ (#1) reveals the dependence on and integration of cutting‐edge editing technologies with traditional transfer platforms. The drought stress theme persists throughout, demonstrating its enduring significance as a fundamental challenge. Gene transformation techniques (including Agrobacterium‐mediated transformation) remain a core technological pillar from the early research stages to the present. Genome editing and RNA interference emerged later, representing the rise of cutting‐edge technologies. The entire diagram illustrates a complete technological evolution driven by fundamental challenges, underpinned by core technologies, and guided by frontier directions.

**FIGURE 11:**
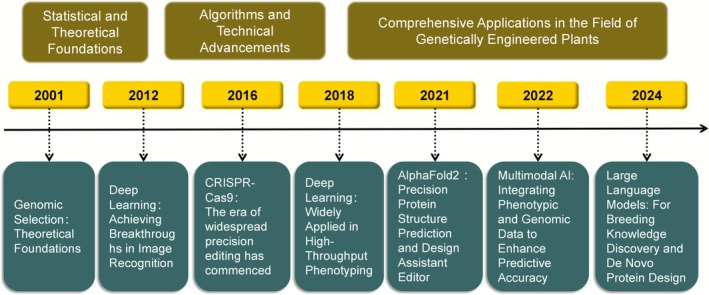
Milestones in the development of artificial intelligence and machine learning in plant genetic engineering.

We have collated and analysed the highly cited keyword literature. The resulting timeline for the development of AI and machine learning in plant genetic engineering is presented in Figure 11. This timeline clearly illustrates the complete evolutionary trajectory of intelligent breeding technologies from theoretical foundations to cutting‐edge applications. This timeline meticulously delineates a coherent and accelerating trajectory of technological advancement. It commences in 2001 with the theoretical proposal of genomic selection, which established the core computational methodology for predicting breeding values using whole‐genome data (Meuwissen et al. 2001; Kredics et al. 2001; Wang et al. 2008). It then progresses to 2012, when breakthroughs in deep learning within image recognition provided powerful tools to address the critical bottleneck of high‐throughput phenotypic analyses (Williams et al. 2012). After 2016, the widespread adoption of CRISPR‐Cas9 technology, which is deeply integrated with phenotypic analyses, propelled breeding from ‘selecting’ natural variation to ‘designing’ ideal variation (Samanta et al. 2016). Since 2021, protein structure prediction tools—such as AlphaFold2, multimodal AI integration techniques, and large language models—have successively entered the breeding domain (Rocafort et al. 2022; Akdel et al. 2022). These methods demonstrate revolutionary potential in rational biomolecular design, multisource data fusion decision‐making, and knowledge discovery with generative design. This evolutionary trajectory indicates that intelligent breeding is founded upon more than two decades of robust and sustained technological advancement. Its developmental trajectory is indicative of a future characterised by data‐driven approaches, model‐assisted decision‐making, and even the deep involvement of artificial intelligence in design.

## 4 Discussion

### 4.1 Technology Trajectory

#### 4.1.1 Shifting From Basic Translational Technologies to Genome Editing

Since gene‐based plant transgenic technology emerged in the last century through 2005, plant gene editing technology has been in its infancy, and the early research was carried out mainly on single genes in model plants such as tobacco through traditional techniques such as the agrobacterium transformation method, gene guns and pollen tubes.

The agrobacterium transformation method was the first instance of plant genetic engineering (Ali et al. 2022). In the late 1970s and early 1980s, scientists reported that *Agrobacterium tumefaciens* (root cancer bacteria) can insert part of its DNA (T‐DNA) into the plant genome (Azizi‐Dargahlou and Pouresmaeil 2024; Huang et al. 2022). This method stably integrates exogenous genes into the plant genome, and the integrated genes can be stably inherited, which provides a reliable means for the study of gene function and genetic improvement of plants (Bomzan et al. 2022). Therefore, the keywords related to representing Agrobacterium rhizogenes and to gene transfer and transgenic plant‐breeding techniques continued to dominate the search concentration in the transgenic plant‐related papers from the late 19th to the early 20th century (Cody et al. 2023; Do et al. 2018). The agrobacterium transformation method was pioneering and central to the early transgenic plant research, promoting the development of plant molecular biology and agricultural biotechnology and laying a solid foundation for the subsequent research (Krenek et al. 2015). As one of the most widely researched plants in the early stage, tobacco is a model plant for studying the mechanisms of gene expression and genetic transformation and provides a technical reference for the transgenic research on other crops (Lu et al. 2024). The successful cultivation of the world's first transgenic plant, tobacco, in 1983 marked the birth of transgenic plant technology and made tobacco the most frequently searched term in the early research stage. As transgenic plant technology has matured, the research focus has shifted from the model plant tobacco to other cash crops (Su et al. 2023). Rice is the staple food for half of the world's population, and many countries have made rice the focus of food security, investing a large amount of money to support the excellent traits of transgenic rice, such as resistance to pests and diseases and stress. These factors have accelerated the breeding process of transgenic rice, which now exceeds that of tobacco and which has become a hotspot for the transgenic plant research (Bhullar and Gruissem 2013).

In 2006, Fire and Mello were awarded the Nobel Prize in Physiology or Medicine for their discovery of the RNAi mechanism, and this technology has proved a powerful tool for studying gene function (Montgomery 2006). From 2006 to 2015, the application of RNAi technology in plant transgenesis became a hotspot (Yu et al. 2016). RNAi can silence genes in cells efficiently and requires only the design of specific RNA fragments without the need for complex vector construction, which greatly simplifies the experimental process (Jin et al. 2021). Moreover, RNAi technology is highly specific, does not rely on genome integration, avoids the risk of insertion mutation, and can silence target genes specifically, reducing the impact on nontarget genes (Krenek et al. 2015). The advantage of being able to simultaneously silence multiple genes makes it possible to study gene families and multigene network regulation (Tang and Galili 2004; Xing et al. 2025). In 2012, Jennifer Doudna et al. analysed the working mechanism of the CRISPR–Cas9 system and demonstrated that it could precisely cut specific DNA using guide RNA (gRNA), indicating the emergence of CRISPR as a programmable gene editing tool (Jinek et al. 2012). In 2013, Ran et al. (2013) applied CRISPR–Cas9 successfully to mammalian and human cells, overcoming the eukaryotic gene editing bottleneck, and promoting the practicality of this technology. From 2015 to the present, the research on gene editing technology applied to transgenic plants has become a major hotspot. As of approximately 2015, genome editing technologies such as ZFN, TALEN and CRISPR–Cas9 were in wide use (Pan et al. 2021). CRISPR–Cas9 technology was used to edit key genes that regulate traits such as yield, quality, disease resistance and herbicide resistance to study their functions and improve crop traits (Barrangou 2023). Homologous recombination, mutations, nucleases, CRISPR Cas9, targeted mutagenesis, guide RNA, endonuclease, gene editing, complex and other keywords closely related to CRISPR technology have increased drastically.

#### 4.1.2 Enhancement of the Application‐Oriented Research

‘Yield’ and ‘crop improvement’ have subsequently become high‐frequency terms, reflecting a shift in the ultimate goal of the research from technology validation to real agricultural problem‐solving. The demands of society for food security, climate change adaptation and nutritional enhancement have driven the research to focus on the use of new technologies to directly improve important agronomic traits in crops (Ali et al. 2022; Feng et al. 2025). The research has accumulated a wealth of gene function data, laying the foundation for the study of yield‐related genes (the maturity of CRISPR and other gene editing technologies), which has made it possible to precisely improve crop yields (Dutta et al. 2023). Moreover, molecular biotechnology, quantitative trait locus (QTL) positioning methods have developed and been continuously improved (Li and Sillanpää 2015), and different locus groups each have their own characteristics. In addition, successive QTL positioning methods have been gradually improved, and several rice yield‐related QTLs, such as Ghd8 and DEP3, have been discovered successively (Powder 2020). The discovery of these QTLs has provided important genetic resources for the genetic improvement of rice yield. At the end of 2023, the Ministry of Agriculture and Rural Development (MARD) issued the first batch of licences for the production and operation of GM maize and soybean seeds, indicating that the industrialisation of GM staple crops has entered the demonstration and promotion stage. Moreover, in the context of food security challenges exacerbated by global population growth and climate change, the surge in searched keywords such as ‘Grain yield’, ‘yield’, ‘Quantitative trait loci’ and ‘Crop improvement’ suggests that grain yield has once again become a key focus for transgenic plants.

Natural variation is an important genetic resource for crop improvement. By studying natural variation within a plant species, scientists can tap into good alleles related to stress resistance, yield and quality (Fernie et al. 2006). Introducing these naturally varying genes into crops through genetic engineering can result in the production of transgenic plants that are adapted to climate change or that have high yields (Krenek et al. 2015). When the evolutionary history of genes is studied, key genes conserved during the evolutionary process, which may be ideal targets for transgenic improvement, can be identified. The popularity of gene editing technologies, such as CRISPR–Cas9, has made the study of natural variation, gene function and transcriptional regulation more efficient (Dutta et al. 2023). The surge in the number of searches for keywords such as natural variation, evolution, activation, mechanisms, pathways, and roles reflects the fact that the research focus in the fields of plant sciences and genetic engineering is shifting to the analysis of gene functions, exploration of molecular mechanisms, use of natural resources and precision regulation. This shift is closely related to the launch and implementation of China's ‘Biobreeding Science and Technology Innovation 2030’ strategy. The strategy identifies the mining of germplasm resources, the analysis of gene regulatory mechanisms and the study of evolutionary adaptations as core breakthrough directions, which directly promote the census and functional mining of natural genetic resources: the development of new gene editing chassis tools and the design of new metabolic pathways, such as the nitrogen fixation module and the regulation of nitrogen utilisation metabolism offered by OsWRKY23–DNR1.

The observed tripartite technological transition (Agrobacterium‐mediated transformation → RNAi multigene silencing → CRISPR eukaryotic editing) aligns with the ‘innovation S‐curve’ predicted by technology lifecycle theory (Borgianni and Rotini 2012). The early‐stage research (1994–2006) focused on proof‐of‐concept validation, characterised by slow adoption rates and incremental improvements (low maturity phase). The 2006–2015 RNAi period marked the ‘growth phase’, with exponential citation growth reflecting paradigm shifts in multigene regulation (Duan et al. 2021; Fazal et al. 2024). Since 2015, CRISPR‐driven precision editing has triggered the ‘maturity phase’, as evidenced by stable citation peaks and diverse applications (e.g., yield enhancement and drought resistance) (Fiaz et al. 2024; Dutta et al. 2023; Sharma and Deutsch 2023). This trajectory underscores the critical role of bibliometric tools in objectively demarcating technology lifecycle stages, which traditional narrative reviews often fail to achieve because of subjective bias.

Moreover, the alignment between policy milestones and research trajectories in China further validates the ‘policy–technology co‐evolution’ hypothesis (Grodal et al. 2023). From 2008 to 2015, the ‘Major Project for Breeding New Varieties of Genetically Modified Organisms’ stimulated the basic research on Agrobacterium transformation and RNAi, which is reflected in the proliferation of publications on the keywords ‘Agrobacterium tumours’ (2006–2012) and ‘drought stress’ (2010–2015) (Liang et al. 2025). From 2016 to 2020, the 13th Five‐Year Plan prioritised rice improvement, which is directly linked to the rice‐centric research boom in 2017–2024 (57.5% of total publications) (Zhuang et al. 2024). From 2021 to the present, the industrialisation of GM maize and soybean (2021–2024) shifted in focus to applied research (e.g., yield and crop improvement). The bidirectional causality of the trajectories described with respect to the ‘policy‐technology co‐evolution’ highlights the critical role of bibliometric tools in objectively delineating the stages of a technology's life cycle, which traditional narrative reviews often fail to do because of subjective biases and the tendency to ignore policy feedback loops.

### 4.2 Analysis of International Cooperation Models

The global transgenic plant research field presents a diversified international cooperation network, with the United States and China as the dual core. By virtue of its profound scientific research accumulation and mature alliance system, the United States has formed a high‐density cooperation network with South Korea, Japan, the United Kingdom and other developed countries in terms of science and technology with close ties and frequent interactions, reflecting the synergistic advantages of traditional scientific and technological powerhouses. In contrast, since 2008, when China launched its national strategy ‘Major Project for the Cultivation of New Genetically Modified Breeds’, China has continued to invest large amounts of R&D resources into this research (Feng et al. 2025), which drove its scientific research output to become the world's largest in 2010, demonstrating a strong endogenous driving force (Zhou et al. 2024; Zuo et al. 2021). Moreover, China has actively implemented an outwards‐looking cooperative strategy, especially when it relies on the ‘Belt and Road’ Science and Technology Innovation Action Plan (Hughes et al. 2020), and has systematically strengthened its in‐depth connections with emerging research regions with great agricultural potential, such as Southeast Asia (e.g., Thailand and Vietnam) and Africa (e.g., Egypt and South Africa). This practice has expanded the breadth and depth of cooperation significantly and led to the active participation of the Asian countries as a whole in the field of transgenics (Liu, Hughes, et al. 2020). Technical export controls, trade frictions and geopolitical factors, such as alliance networks, are significant external drivers of the fluctuations in, or the weakening of, the intensity of bilateral cooperation. The intense geopolitical competition between China and the United States occurs precisely because both countries possess a ‘core’ scale in their scientific research systems, market size and talent reserves. The coexistence of ‘competition’ and ‘interdependence’ manifests as a ‘dual‐core structure’ in real‐world politics. In other words, rather than negating the dual‐core structure, geopolitical tensions underscore the immense gravitational pull and repulsive force between these two cores.

Other important participants, such as New Zealand and Australia, show a pattern of a relative concentration among cooperation recipients and significant regionalisation. Notably, the cooperation participation level is significantly stratified globally. Countries with relatively limited scientific research resources, such as Sudan and Bangladesh, often show a pattern of cooperation mainly through establishing direct and asymmetric connection channels with a single core scientific research country (e.g., the U.S. or China) as the main way to access the proliferation of key technologies and to integrate them into the global R&D network. This practice results in the formation of a typical centre‐to‐periphery dependency structure, which profoundly reveals the highly imbalanced distribution of scientific research resources and the significant regionalisation of the global transgenic research field. These findings reveal differences in the intensity of cooperation connections and outline the mainstream picture and evolutionary trend of international cooperation in this field. China has experienced rapid developments in GM industrialisation, from the start of the GM corn and soybean industrialisation pilot project in 2021 in 20 counties in 5 provinces with yearly expansions, to the first batch of 37 corn and 14 soybean varieties that passed the validation test in 2023, and then the addition of 27 new varieties of corn and 3 new varieties of soybean in 2024 (Mou et al. 2025; Sun, Li, et al. 2024). These developments marked a significant acceleration in both the policy implementation and application levels, which strongly supports and further amplifies China's central position and influence in the global cooperation network.

### 4.3 Perspectives and Future Directions Based on Emerging Trends

#### 4.3.1 Recommendations for Optimising International Cooperation

This study reveals that China and the United States are two pivotal nodes within this research domain. However, the dual‐core structure is not static but is shaped continuously by complex geopolitical dynamics. The future of cooperation between the two nations depends on how they balance competitive imperatives and shared interests. On the basis of the analysis of the structural characteristics and differences in resource allocation of the global international cooperation model for the transgenic plant research, this predicts a strengthening in the strategic synergy between the core nodes (Ahmad et al. 2023). A regular dialogue mechanism must be established between the two major research centres in China and the United States to achieve consensus in key areas such as gene editing technology standards, data sharing and ethical guidelines. Technical barriers should be reduced, and investment duplication should be avoided through joint R&D programmes. In particular, breakthroughs should be made in the mutual recognition of regulatory frameworks to remove obstacles to global industrialisation. To address the resources imbalance in the structure, China should be supported in establishing technology transfer centres in emerging regions such as Southeast Asia and Africa, which rely on the Belt and Road cooperation network, and the United States should be supported through the Consultative Group on International Agricultural Research (CGIAR). Using the resources of the 37 maize or soybean varieties that have been industrialised in China, the country can work with local institutions in other regions to carry out field adaptations and shorten the technology landing cycle. A transnational regulatory coordination working group should be established under the FAO framework to unify the safety assessment process of gene‐edited crops, reduce policy uncertainty and promote the development of transgenic plants globally.

#### 4.3.2 Potential Technical Directions and Outlook of Technology

With the launch and implementation of the ‘Science and Technology Innovation 2030—Agricultural Biological Breeding’ major project, the transgenic plant research may shift in focus to the conservation of germplasm resources and gene mining, the development of gene editing tools for synergistic multigene editing and high‐efficiency delivery, the intelligent design of breeding and synthetic breeding, and the design of new metabolic pathways that do not exist in nature. Notably, the following discussion on the digitisation of germplasm and intelligent breeding is speculative in nature and transcends the direct bibliometric data presented in this study. These concepts are introduced as a logical extrapolation of the data‐intensive trends identified within our analysis of the conventional breeding literature and are intended to stimulate future research directions. Although traditional repositories rely on physical preservation and morphological descriptions (Mandrioli 2023), next‐generation technologies can be used to construct dynamically updated digital twins of germplasm resources by integrating high‐throughput sequencing, phenomics and environmental interactions. For example, the Chinese Academy of Agricultural Sciences (CASA) ‘Strong Seed Technology Initiative’ has initiated the whole‐ genome resequencing of 20 000 core germplasms, combined with an automated collection platform of field phenotypes, to construct a genotype–phenotype association map (GPMA) of rice, wheat and other crops. The goal is to complete the precise positioning of key genes for important traits by 2030, which will increase the efficiency of resource conservation by 50% and provide structured data support for subsequent gene mining. Because the current CRISPR system still has off‐target effects and editing efficiency bottlenecks (Villiger et al. 2024), CRISPR variants could be transformative, such as by transforming Casase through protein engineering to enhance editing precision and adapt diverse delivery vectors (Wang and Doudna 2023). We are now at the beginning of the Breeding 4.0 phase, which is driven by rapid advances in biological big data and informatics technologies. Despite the advances in sequencing technologies that have enabled the decoding of crop genetic variation over the past few decades, the long‐term access to crop growth states for the purpose of high‐throughput phenotypic data collection remains challenging. The keyword explosion analysis reveals a clear trajectory of technological evolution. The foundational transformation era (1990s–2000s) experienced an early explosion dominated by terms such as agrobacterium, particle bombardment, protoplasts, and transgenic plants, marking the ground‐laying phase of plant genetic engineering. The focus is on establishing reliable gene transfer and regeneration techniques. Subsequently, the 2010s witnessed specialised precision control. Keywords such as CRISPR–Cas9, targeted mutagenesis, gene editing, and guide RNA have emerged. This emergence marks a pivotal transition from random gene insertion to precise genome engineering. From 2018 to the present, the focus has been on trait‐directed and data‐driven breeding. Crucially, the most recently prevalent keywords (2019–2024) no longer focus on technical methods but have shifted towards outcomes and complexity. Examples include gene editing (the most powerful burst at 61.2), yield, quantitative trait loci (QTL), natural variation and crop improvement. Upon consulting the literature associated with these keywords, we discovered that this evolution establishes the ideal foundation for the adoption of AI/ML. The advent of gene editing has given rise to the necessity of predicting optimal editing targets (Sun, Lei, et al. 2024). The emphases on complex traits, such as yield, and quantitative trait loci necessitate the analysis of substantial, multiomics datasets to understand natural variation (Kim et al. 2024; Sojka et al. 2024). These challenges, namely, predictive modelling and the mining of complex biological data, represent the core strengths of artificial intelligence and machine learning (Yang et al. 2023).

The technological evolutionary trajectory provides a clear answer to the fundamental question of why AI/ML represents the future of breeding. The timeline demonstrates that the driving forces within the breeding sector have shifted from traditional ‘empirical discovery’ to systematic ‘data‐driven’ and ‘intelligent design’ approaches. Early statistical methods, exemplified by genomic selection, addressed the challenge of predicting phenotypes from genotypes (Desta and Ortiz 2014; Piyasatian et al. 2007). However, such research encountered bottlenecks in analysing high‐dimensional, nonlinear relationships (Sinervo and Svensson 2002). This development naturally precipitated the advent of more sophisticated machine‐learning algorithms, particularly those of the deep‐learning variety (Jubair and Domaratzki 2022). By overcoming the critical bottleneck of acquiring high‐throughput phenotypes, these methods have resulted in a substantial expansion of the dimensions and scale of the data available for modelling (Williams et al. 2012; Alemu et al. 2024). The advent of gene editing technologies empowered the proactive creation of variation, propelling a relentless pursuit of enhancing editing efficiency and functional prediction (Montesinos‐López et al. 2024). This pursuit has led to the emergence of rational design tools such as AlphaFold2, which leverages artificial intelligence (AI) for structural predictions (Shi et al. 2025). Confronting the hypercomplex ‘data universe’ generated by multiomics, phenotypes, and environmental interactions, large language models (LLMs) have emerged that are capable of integrating multimodal information and of reasoning (Gao et al. 2023; Crossa et al. 2025; Lam et al. 2024; Yoosefzadeh‐Najafabadi 2025). This interconnected sequence of events illustrates that the encroachment of AI does not represent an externally imposed phenomenon; rather, it is an inherent and unavoidable technological necessity that has emerged as a consequence of the relentless pursuit of enhanced precision and efficiency within the domain of scientific breeding. The fusion of machine learning with AI macromodelling; the clustering of robots in the field; and metabolic pathway reprogramming technologies, which utilise computational simulation and enzyme engineering to design unnatural pathways and genetically isolated vectors, present promising pathways. They are likely to result in the design of materials that transcend naturally evolved matter, thereby creating entirely new biological systems.

## 5 Conclusion

In this study, the overall situation, technological evolution, future development direction and optimisation path of the global transgenic plant research are reviewed. In terms of the research network, a diversified cooperation pattern has been formed with China and the United States as the nucleus and with the United States and Korea as well as Japan and the United Kingdom constituting high‐density cooperation clusters. Since the launch of the major special project on transgenics in China in 2010, the number of scientific research papers issued has increased drastically to occupy first place globally. The ‘One Belt and One Road’ has deepened its connection with emerging agricultural regions such as Southeast Asia and Africa and strengthened its global influence by accelerating the industrialisation process. Global influence has been strengthened by this acceleration. This situation has also arisen because of biotechnology development, for which the basic transformation period of 1994–2006 had Agrobacterium‐mediated tobacco transgenic technology as its core; the technological breakthrough period of 2006–2015 focused on RNAi to promote the study of multigene silencing and the CRISPR–Cas9 system to overcome the bottleneck of eukaryotic editing; and the deepened application period of 2015–present day, during which the research focused on food security. In this process, CRISPR technology drives the related research, and QTL localisation and natural variation mining help improve complex agronomic traits. On the basis of continuous maturation of genetic engineering and the strategy of ‘Biobreeding Science and Technology Innovation 2030’, we focus on the four major directions of germplasm resource digitisation, breakthroughs in multigene editing, the convergence of intelligent breeding, and innovations in synthetic biology to promote the future industrialisation and development of transgenic plant technology.

Despite the robustness of our bibliometric approach, certain limitations remain. The following four are worth discussing. First, our reliance on the Web of Science (WoS) core collection, although the standard for bibliometric studies, presents a well‐documented bias towards English‐language publications. This bias means that our analysis captures primarily global core knowledge production and may underrepresent systematically the research from regions where findings are often disseminated in local languages or through national databases. Consequently, conclusions about geographic trends and the relative prominence of certain research topics should be interpreted in light of this Anglophone‐centric bias. Second, keyword bias arises whereby automated clustering algorithms may overlook subtle conceptual shifts and confuse terms such as ‘CRISPR’ and ‘gene editing’ into a single category. Third, static analyses have limitations. While this study captured cross‐sectional collaboration patterns, it lacked any longitudinal tracking of individual researcher mobility and funding flows. Future research should integrate Scopus/PubMed data, use machine learning for a semantic text analysis and combine bibliometrics with patent data to map technology transfer pathways. Finally, the search strategy utilised by the present study concentrated on well‐established breeding techniques; however, it did not encompass the latest terminology related to artificial intelligence and digital tools. The present study employed a focused, bibliometric retrieval strategy; consequently, it did not encompass all of the terminology within the fields of artificial intelligence and machine learning (such as ‘deep learning’, ‘neural networks’, and ‘large language models’). Consequently, the present analysis could not quantify directly emerging trends within the intelligent breeding domain. The study predictions should be understood as a qualitative prediction based on a contextual analysis of the high‐impact literature and an emerging field consensus rather than as a direct quantitative conclusion of this research. Future bibliometric studies could incorporate these terms explicitly to track the rise of these fields quantitatively.

## Author Contributions

**Tongxiao Xu:** writing – original draft preparation, visualisation, methodology; **Teng Wang:** visualisation, methodology; **Cong Zhang:** formal analysis; **Yuan Cao** and **Xiaoyun He:** writing – review and editing. All authors contributed to manuscript revision, read and approved the submitted version. All authors have read and agreed to the published version of the manuscript.

## Funding

This work was supported by Science and Technology Innovation 2030‐Biological Breeding Major Projects (2023ZD0406310).

## Conflicts of Interest

The authors declare no conflicts of interest.

## Supporting information


**Appendix S1:** pbi70550‐sup‐0001‐AppendixS1.docx.

## Data Availability

Bibliometric data were obtained from the Web of Science Core Collection (WoSCC). Detailed retrieval strategies, keywords, and inclusion criteria are provided in the supplementary methods section and dataset https://www.scidb.cn/s/aAVJZr, facilitating dataset replication or further exploration. The authors confirm that the data supporting the findings of this study are available within the Appendix S1 and from the corresponding author.
