# Supplementary material for: Bibliometric‐Based Analysis of Global Trends and Collaborative Networks in Plant Genetic Engineering (1994–2024)
Source: Plant Biotechnol J. 2026 Jan 19;24(5):3065–83. doi: 10.1111/pbi.70550 (PMC13110155; doi:10.1111/pbi.70550)
Supplement: Supplementary file 1 — Appendix S1: pbi70550‐sup‐0001‐AppendixS1.docx. [file PBI-24-3065-s001.docx]

**Bibliometric-based analysis of global trends and collaborative networks in transgenic plant technology （1994-2024）**

**Supplymentary mateirals**

**Supplementary Materials and methods**

**Search strategy**

(TI=("gene gun" OR "particle bombardment" OR "micro bullet technology" OR "high-velocity particle microprojection" OR "pollen tube pathway" OR "microinjection" OR "micro perfusion technology" OR "gene microinjection" OR "embryonic stem cells" OR "ES cells" OR "sperm vector" OR "somatic cell nuclear transfer" OR "agrobacterium-mediated transformation" OR "tumour inducing plasmid" OR "T-DNA" OR "viral vector" OR "lenti viral vector" OR "rAAV" OR "retroviral vector" OR "retrovirus vector" OR "adenovirus vectors" OR "adenoviral vector" OR "herpesvirus vector" OR "adeno-associated virus" OR "electroporation" OR "electrotransfection" OR "electroosmosis" OR "liposomes" OR "calcium phosphate co precipitation" OR "PEG" OR "CRISPR" OR "Cas" OR "gene knock out" OR "gene knock in" OR "zinc finger nucleases" OR "ZFNs" OR "transcription activator-like effector nucleases" OR "TALEN" OR "base editing" OR "prime editing" OR "gene silencing" OR "RNA interference" OR "RNAi" OR "siRNA" OR "shRNA" OR "TGS" OR "PTGS" OR "genome editing" OR "co-transformation")

AND

TI=("Corn*"OR "Maize*" OR "Corn Oil*" OR "Maize Oil*" OR "Corn Flour*" OR "Cornstarch*" OR "Cornmeal*" OR "soybean*" OR "soybean meal*" OR "Soybean Oil*" OR "Soy flour*" OR "cotton*" OR "Genetic cotton*" OR "Cotton seed*" OR "Corn*"OR "Maize*" OR "Corn Oil*" OR "Maize Oil*" OR "Corn Flour*" OR "Cornstarch*" OR "Cornmeal*" OR "rice*" OR "GMP rice*" OR "tomato*" OR "Tomato seed*" OR "Tomato sauce*" OR "wheat*" OR "barley*" OR "hordeum*" OR "hordeum vulgare*" OR "potato*" OR "irish potato*" OR "white potato* OR cucurbita*" OR "cucurbita moschata*" OR "cushaw*" OR "pumpkin*" OR "squash*" OR "winter squash*"OR"peppers*" OR "chili* OR cowpea*" OR "Transgenic bean*" OR "bean*" OR "common bean*" OR "french bean*" OR "kidney bean*" OR "snap bean* OR Chinese sorghum*" OR "durra*" OR "grass sorghum*" OR "kaoliang*" OR "sorghum*" OR "sorghum bicolor*" OR "sorghum seed*" OR "snap bean* OR Brassica* OR bird rape*" OR "canola*" OR "napus*" OR "oilseed rape*" OR "rape*" OR "rape seed*" OR "rapeseed*" OR "rapeseed oil* OR mustard*" OR "Genetic mustard*" OR "Brassica oleracea*" OR "cabbage*" OR "common cabbage*" OR "head cabbage*" OR "kohlrabi*" OR "wild cabbage*" OR "kale*" OR "broccoli*" OR "cauliflower*" OR "brussels sprouts* OR blackberry" OR "blackberries OR cyperus rotundu*" OR "Thlaspi arvense*" OR "Transgenic cyperus rotundu*" OR "banana*" OR "banana fruits*" OR "banana plantation*" OR "common banana*" OR "dwarf banana*" OR "musa* OR chinese walnut*" OR "juglans*" OR "juglans regia*" OR "persian walnut*" OR "walnut*" OR "Genetically rigen walnut* OR apple*" OR "apple cider*" OR "apple orchard*" OR "apple tree* OR alfalfa*" OR "alfalfa cultivars*" OR "common alfalfa*" OR "medicago sativa*" OR "medicago sativa l* OR eggplant*" OR "Genetic eggplant* OR cassava*" OR "cassava-based" OR "manihot esculenta* OR pineapple* OR sugarcane* OR rye grass*" OR "Transgenic ryegrass*")

AND

TI=("transgenic*" OR "gene editing" OR "genome editing" OR "genetic engineering" OR "genetically modified" OR "GMO" OR "recombinant DNA" OR "GM crop*") )

OR

(AB=("gene gun" OR "particle bombardment" OR "micro bullet technology" OR "high-velocity particle microprojection" OR "pollen tube pathway" OR "microinjection" OR "micro perfusion technology" OR "gene microinjection" OR "embryonic stem cells" OR "ES cells" OR "sperm vector" OR "somatic cell nuclear transfer" OR "agrobacterium-mediated transformation" OR "tumour inducing plasmid" OR "T-DNA" OR "viral vector" OR "lenti viral vector" OR "rAAV" OR "retroviral vector" OR "retrovirus vector" OR "adenovirus vectors" OR "adenoviral vector" OR "herpesvirus vector" OR "adeno-associated virus" OR "electroporation" OR "electrotransfection" OR "electroosmosis" OR "liposomes" OR "calcium phosphate co precipitation" OR "PEG" OR "CRISPR" OR "Cas" OR "gene knock out" OR "gene knock in" OR "zinc finger nucleases" OR "ZFNs" OR "transcription activator-like effector nucleases" OR "TALEN" OR "base editing" OR "prime editing" OR "gene silencing" OR "RNA interference" OR "RNAi" OR "siRNA" OR "shRNA" OR "TGS" OR "PTGS" OR "genome editing" OR "co-transformation")

AND

AB=("Corn*"OR "Maize*" OR "Corn Oil*" OR "Maize Oil*" OR "Corn Flour*" OR "Cornstarch*" OR "Cornmeal*" OR "soybean*" OR "soybean meal*" OR "Soybean Oil*" OR "Soy flour*" OR "cotton*" OR "Genetic cotton*" OR "Cotton seed*" OR "Corn*"OR "Maize*" OR "Corn Oil*" OR "Maize Oil*" OR "Corn Flour*" OR "Cornstarch*" OR "Cornmeal*" OR "rice*" OR "GMP rice*" OR "tomato*" OR "Tomato seed*" OR "Tomato sauce*" OR "wheat*" OR "barley*" OR "hordeum*" OR "hordeum vulgare*" OR "potato*" OR "irish potato*" OR "white potato* OR cucurbita*" OR "cucurbita moschata*" OR "cushaw*" OR "pumpkin*" OR "squash*" OR "winter squash*"OR"peppers*" OR "chili* OR cowpea*" OR "Transgenic bean*" OR "bean*" OR "common bean*" OR "french bean*" OR "kidney bean*" OR "snap bean* OR Chinese sorghum*" OR "durra*" OR "grass sorghum*" OR "kaoliang*" OR "sorghum*" OR "sorghum bicolor*" OR "sorghum seed*" OR "snap bean* OR Brassica* OR bird rape*" OR "canola*" OR "napus*" OR "oilseed rape*" OR "rape*" OR "rape seed*" OR "rapeseed*" OR "rapeseed oil* OR mustard*" OR "Genetic mustard*" OR "Brassica oleracea*" OR "cabbage*" OR "common cabbage*" OR "head cabbage*" OR "kohlrabi*" OR "wild cabbage*" OR "kale*" OR "broccoli*" OR "cauliflower*" OR "brussels sprouts* OR blackberry" OR "blackberries OR cyperus rotundu*" OR "Thlaspi arvense*" OR "Transgenic cyperus rotundu*" OR "banana*" OR "banana fruits*" OR "banana plantation*" OR "common banana*" OR "dwarf banana*" OR "musa* OR chinese walnut*" OR "juglans*" OR "juglans regia*" OR "persian walnut*" OR "walnut*" OR "Genetically rigen walnut* OR apple*" OR "apple cider*" OR "apple orchard*" OR "apple tree* OR alfalfa*" OR "alfalfa cultivars*" OR "common alfalfa*" OR "medicago sativa*" OR "medicago sativa l* OR eggplant*" OR "Genetic eggplant* OR cassava*" OR "cassava-based" OR "manihot esculenta* OR pineapple* OR sugarcane* OR rye grass*" OR "Transgenic ryegrass*")

AND

AB=("transgenic*" OR "gene editing" OR "genome editing" OR "genetic engineering" OR "genetically modified" OR "GMO" OR "recombinant DNA" OR "GM crop*") )
